# Prognostic value of baseline metabolic tumour volume in advanced-stage Hodgkin’s lymphoma

**DOI:** 10.1038/s41598-021-02734-w

**Published:** 2021-12-01

**Authors:** Pierre Pinochet, Edgar Texte, Aspasia Stamatoullas-Bastard, Pierre Vera, Sorina-Dana Mihailescu, Stéphanie Becker

**Affiliations:** 1 Nuclear Medicine Department, Henri Becquerel Cancer Centre, Rue d’Amiens, Rouen, France; 2 QuantiF-LITIS (EA 4108-FR CNRS 3638), Faculty of Medicine, University of Rouen, Rouen, France; 3 Hematology Department, Henri Becquerel Cancer Centre, Rouen, France; 4 INSERM U1245, Henri Becquerel Cancer Centre, Rouen, France; 5 Department of Statistics and Clinical Research Unit, Henri Becquerel Cancer Centre, Rouen, France

**Keywords:** Hodgkin lymphoma, Positron-emission tomography

## Abstract

Our aim was to evaluate the prognostic value of initial total metabolic tumour volume (TMTV) in a population of patients with advanced-stage Hodgkin’s lymphoma (HL). We retrospectively included 179 patients with stage IIb-III-IV Hodgkin’s disease who received BEACOPP or ABVD as the first-line treatment. The initial TMTV was determined using a semi-automatic method for each patient. We analysed its prognostic value in terms of 5-year progression-free survival (PFS), overall survival, and positron emission tomography (PET) response after two courses of chemotherapy. Considering all the treatments and using a threshold of 217 cm^3^, TMTV was predictive of 5-year PFS and PET response after two courses of chemotherapy. In multivariable analysis involving TMTV, IPI score, and the first treatment received, TMTV remained a baseline prognostic factor for 5-year PFS. In the subgroup of patients treated with BEACOPP with a threshold of 331 cm^3^, TMTV was predictive of PET response, but not 5-year PFS (*p* = 0.087). The combined analysis of TMTV and PET response enabled the individualisation of a subgroup of patients (low TMTV and complete response on PET) with a very low risk of recurrence. Baseline TMTV appears to be a useful independent prognostic factor for predicting relapse in advanced-stage HL in ABVD subgroup, with a tendency of survival curves separation in BEACOPP subgroup.

## Introduction

Many patients with Hodgkin’s lymphoma (HL) are treated with standard therapeutics and are, therefore, at risk of potential long-term complications. However, approximately 15% of patients relapse within 5 years in the advanced stages^1^. Prognostic factors are necessary to identify patients at low or high risk to avoid relapse in cases of insufficient treatment or long-term toxicity for intense chemotherapy regimens.

Many prognostic factors have been identified in localised stage Hodgkin’s disease, (including bulky tumour, number of regions involved, B-symptoms, erythrocyte sedimentation rate, advanced age), and allow the initial treatment to be adapted^2^. In contrast, in the advanced stages of HL, tumour bulk and other prognostic factors are less predictive of survival^3^. A prognostic score has been developed for these patients by the International Prognostic Factor Project (IPS score)^3^, but it is not used to adapt the treatment.

^18^F-fluorodeoxyglucose positron emission tomography (FDG-PET) scanning is widely used in the diagnosis and therapeutic evaluation of lymphoma, and in particular, it allows different response-adapted treatment strategies after two courses of chemotherapy^4,5^. However, a pretherapeutic prognostic factor is necessary to achieve a risk-stratified treatment.

One of the advantages of initial FDG-PET imaging is the evaluation of the total metabolic tumour volume (TMTV) in relation to tumour size, tumour activity, and the tumour microenvironment. The TMTV measured on an initial PET scan has been shown to be an independent prognostic factor in localised Hodgkin's disease^6^. In advanced stages, one study has shown that TMTV is a predictor of early response after two cycles of bleomycin, etoposide, doxorubicin, cyclophosphamide, vincristine, procarbazine, and prednisone. However, the prognostic value of TMTV in progression-free survival (PFS) has not been highlighted^7^.

Therefore, our study aimed to investigate the prognostic value of TMTV for 5-year PFS in patients with advanced Hodgkin's disease treated with ABVD (doxorubicin, bleomycin, vinblastine, and dacarbazine)—or BEACOPP (bleomycin, etoposide, doxorubicin, cyclophosphamide, vincristine, procarbazine, and prednisone) -based chemotherapy. The TMTV prognostic value was compared with the IPS score and early PET response after two courses of chemotherapy.

## Materials and methods

### Patients

This retrospective monocentric study included patients aged ≥ 16 years with histologically proven HL and advanced stage Ann Arbor (IIb, III, and IV), confirmed by the haematology local committee, treated in first line with 6 or 8 cycles of ABVD regimen or 2 to 6 cycles of BEACOPP regimen. Patients whom therapy was de-escalated from BEACOPP to ABVD were analysed in the BEACOPP group. All FDG-PET/CT were performed at the Henri Becquerel Center (Rouen, France) between March 2006 and December 2017.

For refractory or relapsed patients, salvage chemotherapy with DHAP (cisplatin, cytarabine, and dexamethasone) or ICE (ifosfamide, carboplatin and etoposide) regimen were proposed.

Patients with nodular lymphocyte predominant lymphoma or other concomitant diseases with ^18^F-FDG avidity were excluded.

Clinical data on the following variables were obtained from all the patients: age at disease onset, sex, Eastern Cooperative Oncology Group (ECOG) performance status, Ann Arbor staging system, results of osteomedullary biopsy, presence of B symptoms, and mediastino-thoracic index. The IPS score was calculated for each patient.

The study was approved by the Institutional Review Board (no. 2102B).

### PET acquisition and interpretation

PET/CT scans were acquired on three different PET/CT systems—Biograph 16 (Siemens Healthcare, Erlangen, Germany), Discovery 710 (GE Healthcare, Chicago, Illinois, United States), and Biograph mCT (Siemens Healthcare, Erlangen, Germany). All subsequent PET/CT scans conducted for treatment evaluation were performed using the same PET/CT device that was used for the baseline scan.

Patients fasted for at least 6 h before the 18F-FDG injection. Injection was not administered unless the glucose blood level was < 1.8 g/L. The activity of the injected ^18^F-FDG activity ranged from 3.5 MBq/Kg to 4.5 MBq/kg, with a maximum activity of 450 MBq. Scans were acquired approximately 60 min after the injection. CT scans were acquired from the orbits to the midthigh in most cases and whole-body acquisition was conducted in others, with 120 kV and 100–150 mAs (based on the patient’s weight). OSEM reconstruction was performed with routine parameters (two iterations and 24 subsets). Contrast media injections were not administered.

The response to PET2 was evaluated by using the Deauville score and modified Deauville score (2011 AHL criteria taking into account 140% of liver background)^4^.

### Segmentation

We analysed TMTV using the Beth Israel plugin for FIJI (ImageJ), a shareware from the Beth Israel Deaconess Medical Center, Division of Nuclear Medicine and Molecular Imaging^8^.

Each hypermetabolic focus suspected of lymphomatous localisation was segmented on fused PET/CT images with a threshold of 41% of SUVmax. First, segmentation was performed automatically using the software. Manual verification was then performed with, if necessary, the addition of potential forgotten foci and modification of the automatically segmented ones.

Segmentation of the hypermetabolic lymph nodes, spleen, bone, and other anatomical foci was performed independently, and the TMTV and total lesion glycolysis (TLG) were recorded for each of them.

The TMTV was obtained by summing the metabolic volumes of all nodal and extranodal lesions. Bone marrow was involved in the volume measurement only if focal uptake was observed. The spleen was considered as involved if there was focal uptake or diffuse uptake higher than 150% of the liver background, as recommended^9^.

Three nuclear physicians (SB, ET, and PP) performed the segmentations, with each patient’s foci segmented by two of the physicians. For the two values of TMTV and TLG obtained for each patient, the reference value retained was the one determined by the most experienced observer.

### Statistical analysis

Statistical analysis was performed using the R software, version 4.0.4^10^. Continuous data were compared using independent samples *t-tests*. Agreement between two observers was evaluated by using intraclass correlation coefficient (ICC) to measure the consistency of MTV and TLG evaluations. The 95% confidence intervals of ICC were estimated using 10,000 bootstrap replications with the adjusted bootstrap percentile^11,12^. The median follow-up was calculated using the reverse Kaplan–Meier method^13^. PFS and OS were estimated from the date of diagnosis to progression (first clinical suspicion of recurrence or diagnosis of recurrence on computed tomography (CT) or positron emission tomography (PET)) or death, and death, respectively. The statistical analysis was performed at 5 years; hence, the data was censored at this time. Receiver operating characteristic (ROC) curves were used to predict the PFS at 5 years for each segmentation method by identifying the optimal cut-off values. Survival probabilities were calculated using the Kaplan–Meier method. Log-rank tests and multivariate analyses were performed using Cox models. Statistical significance was set at a two-tailed *p* value of < 0.05. For secondary analyses, a Hochberg correction was applied to control the risk of family-wise type I error at 5%^14^.

### Ethical approval

This study was performed in accordance with the Declaration of Helsinki and local laws, and the protocol was approved by the Institutional Review Board of Henri Becquerel Centre (n°2102 B).

### Informed consent

The institutional review board of Henri Becquerel Centre waived the need to obtain inform consent.

## Results

### Patients’ characteristics

A total of 179 patients were included in the study. The median follow-up period was 5.43 years (4.99–6.03 years). The mean 5-year PFS and OS were 70% (range 63.3–77.3%) and 87% (range 81.7–92.5%), respectively.

Sixty-four (35.8%) patients were treated with BEACOPP, and 115 (64.2%) patients were treated with ABVD. Patients initially treated with BEACOPP and subsequently with ABVD for secondary de-escalation were classified into the BEACOPP group. Among the 179 patients, 110 (61.5%) underwent intermediate two-cycle PET scan (PET2): 62 patients were treated with BEACOPP and 48 with ABVD. The patient characteristics are presented in Table 1.

**Table 1:** Baseline patient characteristics.

Characteristic	Overall, N = 179	ABVD, N = 115	BEACOPP, N = 64	*p* value
**Age**				< 0.0001
Mean ± standard deviation (SD)	39.77 ± 15.46	43.15 ± 16.51	33.70 ± 11.14	
Median (25%; 75%)	37.00 (26.00; 51.00)	41.00 (28.50; 54.00)	32.50 (24.00; 41.00)	
**Sex**				0.44
Female	66 (36.9%)	40 (34.8%)	26 (40.6%)	
Male	113 (63.1%)	75 (65.2%)	38 (59.4%)	
Sex ratio (M:F)	1.71	1.88	1.46	
**Binarised Eastern Cooperative Oncology Group performance status**				1
0–2	172 (97.2%)	110 (97.3%)	62 (96.9%)	
3–4	5 (2.8%)	3 (2.7%)	2 (3.1%)	
**Ann Arbor staging**				0.07
IIb bulky	2 (1.1%)	2 (1.7%)	0 (0.0%)	
III	73 (40.8%)	53 (46.1%)	20 (31.2%)	
IV	104 (58.1%)	60 (52.2%)	44 (68.8%)	
**Osteo-medullary biopsy**				0.005
Negative	139 (80.3%)	96 (88.1%)	43 (67.2%)	
Positive	20 (11.6%)	9 (8.3%)	11 (17.2%)	
Not performed	9 (5.2%)	3 (2.8%)	6 (9.4%)	
Not interpretable	5 (2.9%)	1 (0.9%)	4 (6.2%)	
**B symptoms**				0.31
Yes	86 (48.0%)	52 (45.2%)	34 (53.1%)	
No	93 (52.0%)	63 (54.8%)	30 (46.9%)	
**Mediastino-thoracic index (MTI)**				0.16
Not bulky(MTI < 0.35)	147 (82.1%)	91 (79.1%)	56 (87.5%)	
Bulky (MTI ≥ 0.35)	32 (17.9%)	24 (20.9%)	8 (12.5%)	
**Binarized international prognostic score (IPS)**				0.62
0–2	73 (41.5%)	48 (42.9%)	25 (39.1%)	
3–7	103 (58.5%)	64 (57.1%)	39 (60.9%)	
**PET0 parameters**				
**SUVmax**				0.64
Mean ± SD	14.71 ± 6.34	14.54 ± 7.09	15.01 ± 4.75	
Median (25%; 75%)	14.00 (11.29; 17.34)	13.00 (10.95; 16.92)	15.01 (11.74; 17.70)	
**TMTV (cm**^**3**^**)**				0.005
Mean ± SD	346.72 ± 348.37	292.98 ± 265.46	443.29 ± 447.92	
Median (25%; 75%)	251.06 (125.57; 392.37)	217.30 (120.92; 339.30)	306.98 (169.30; 473.97)	
**TLG (cm**^**3**^**)**				0.01
Mean ± SD	2200.03 ± 2643.06	1838.05 ± 1981.79	2850.45 ± 3458.58	
Median (25%; 75%)	1389.24 (595.12; 2507.36)	1325.61 (514.05; 2286.91)	1624.07 (765.86; 3128.74)	

### Inter-observer correlation

ICC (intraclass correlation coefficient) between observer 1 (SB) and observer 2 (ET) (59 patients) was 0.92 (0.73–0.98) for TMTV and 0.92 (0.77–0.98) for TLG. ICC between observer 1 (SB) and observer 3 (PP) (59 patients) was 0.93 (0.67–0.98) for TMTV and 0.97 (0.77–1.00) for TLG. The values of ICC between observer 2 (ET) and observer 3 (PP) (61 patients) were 0.91 (0.77–0.97) or TMTV and 0.97 (0.92–0.99) for TLG. All the ICC values were excellent (> 0.90)^15^.

### Baseline PET parameters for the whole population

**Figure 1:**
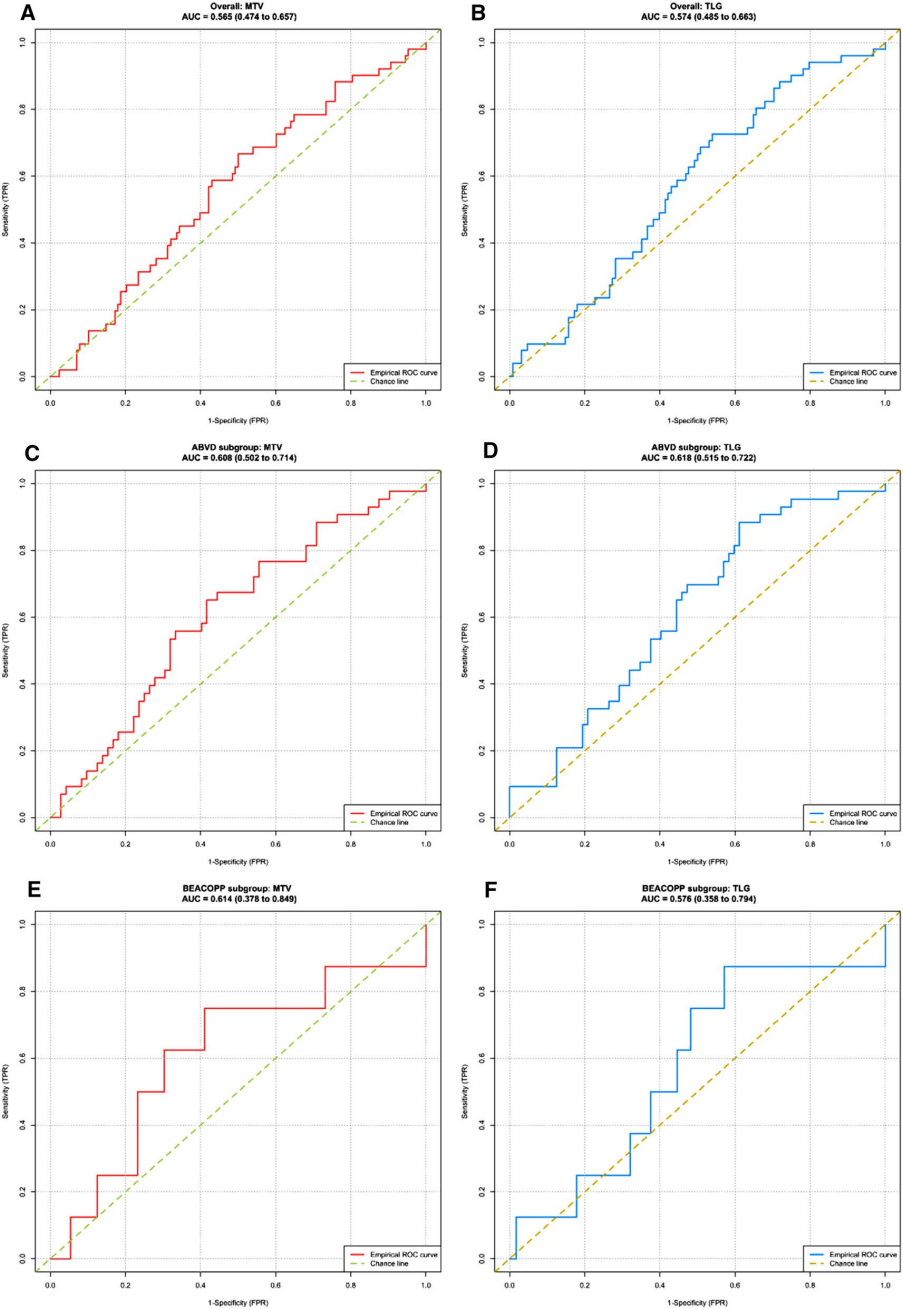
Receiver operating characteristics (ROC) curves analysing the prognostic performance of total metabolic tumour volume (TMTV) and TLG on 5-year progression-free survival.

**Figure 2:**
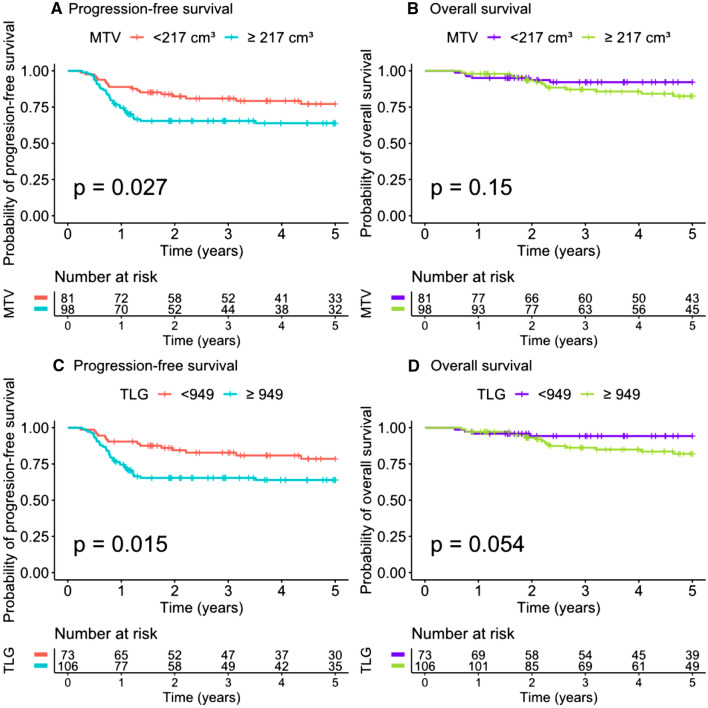
Kaplan Meier analysis of 5-year progression free survival and overall survival for total metabolic tumour volume (TMTV) and TLG (whole population).

Median TMTV was 251.06 cm^3^ (range 125.58–392.37). The ROC curve analysis of the prognostic performance of TMTV on 5-year PFS showed an AUC of 0.57. Using the Youden index, the best TMTV cut-off value was 217 cm^3^ for 5-year PFS, with a sensitivity of 67% and a specificity of 50% (Fig. 1). The presence of a TMTV ≥ 217 cm^3^ was associated with a significantly shorter PFS (*p* = 0.027) and a hazard ratio (HR) of 1.91 (1.07 to 3.42). The 98 patients with a high TMTV had a significantly worse outcome, with a 5-year PFS of 64% vs. 77% for patients with a lower TMTV. Using the same cut-off value, the presence of a high TMTV was not significantly associated with a shorter OS (*p* = 0.15) (Fig. 2).

Median TLG was 1389.24 (range 595.13–2507.36). The ROC curve analysis of the prognostic performance of TLG on 5-year PFS showed an AUC of 0.57. Using the Youden index, the best TLG cut-off value was 949 for 5-year PFS, allowing a sensitivity of 73% and a specificity of 46% (Fig. 1). The presence of a TLG ≥ 949 was associated with a significantly shorter PFS (*p* = 0.015) and a HR of 2.11 (1.14–3.91). The 106 patients with a high TLG had a significantly worse outcome, with a 5-year PFS of 64% vs. 79% for patients with a lower TLG. Using the same cut-off value, the *p* value evaluating the association between TLG and OS was also provided, but caution is to be used when interpreting this value because the hypothesis of proportional hazard was not respected (Fig. 2).

#### Subgroup analysis

While separating the sample according to treatment, the optimal cut-off for TMTV in ABVD subgroup remained the same as in overall sample (217 cm^3^), with a sensitivity of 65% and a specificity of 58%.

**Figure 3:**
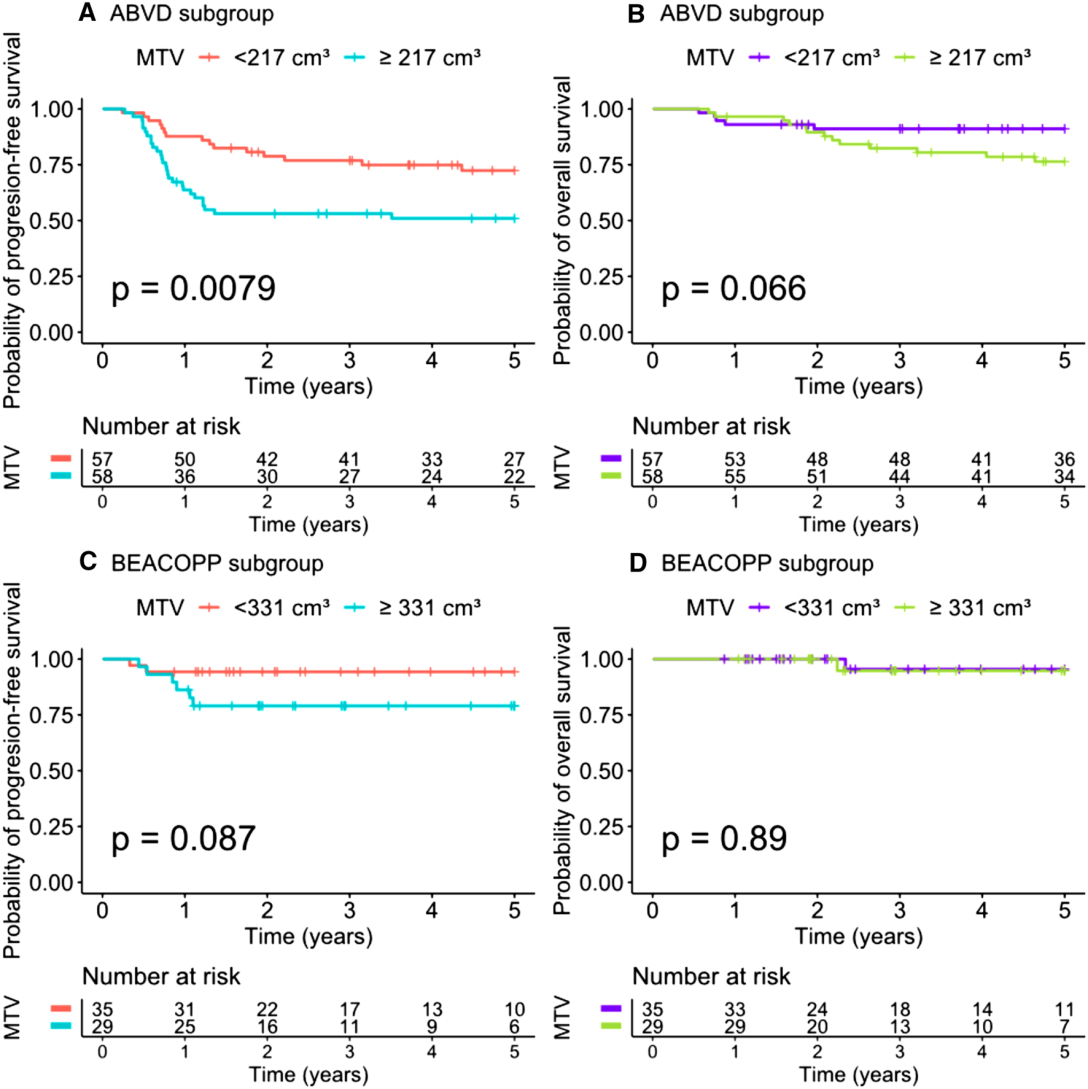
Kaplan Meier analysis of 5-year progression-free survival in relation to total metabolic tumour volume (TMTV) according to treatment.

TMTV remained predictive of 5-year PFS (*p* = 0.0079) with an HR of 2.29 [1.22–4.3]. The 58 patients with a high TMTV had a significantly worse outcome, with a 5-year PFS of 51% vs. 72% for patients with a lower TMTV (Fig. 3).

In the BEACOPP subgroup, the optimal cut-off for TMTV was of 331 cm^3^, with a sensitivity of 75% and a specificity of 59%. TMTV was not predictive of 5-year PFS, although a tendency of survival curves separation is observed (*p* = 0.087) with a HR of 3.68 [0.74 to 18.3]. The patients with a high TMTV had a 5-year PFS of 79%, compared to 94% in patients with a low MTV (Fig. 3).

Patients in the BEACOPP subgroup had a significantly higher 5-year PFS than those in the ABVD subgroup (*p* = 0.0017), with a HR of 0.32 (0.15–0.68). The 115 patients in the ABVD subgroup had a significantly worse outcome than patients in the BEACOPP group (PFS of 62% vs. 87%).

### PET2 response

Of the 110 patients who underwent PET2, the modified Deauville score was predictive of 5-year PFS (*p* = 0.048) with a HR of 2.34 (0.98–5.58). The 24 patients with a positive PET2 had a slightly significantly worse outcome, with a 5-year PFS of 67% vs. 82% for patients with a negative one.

**Figure 4:**
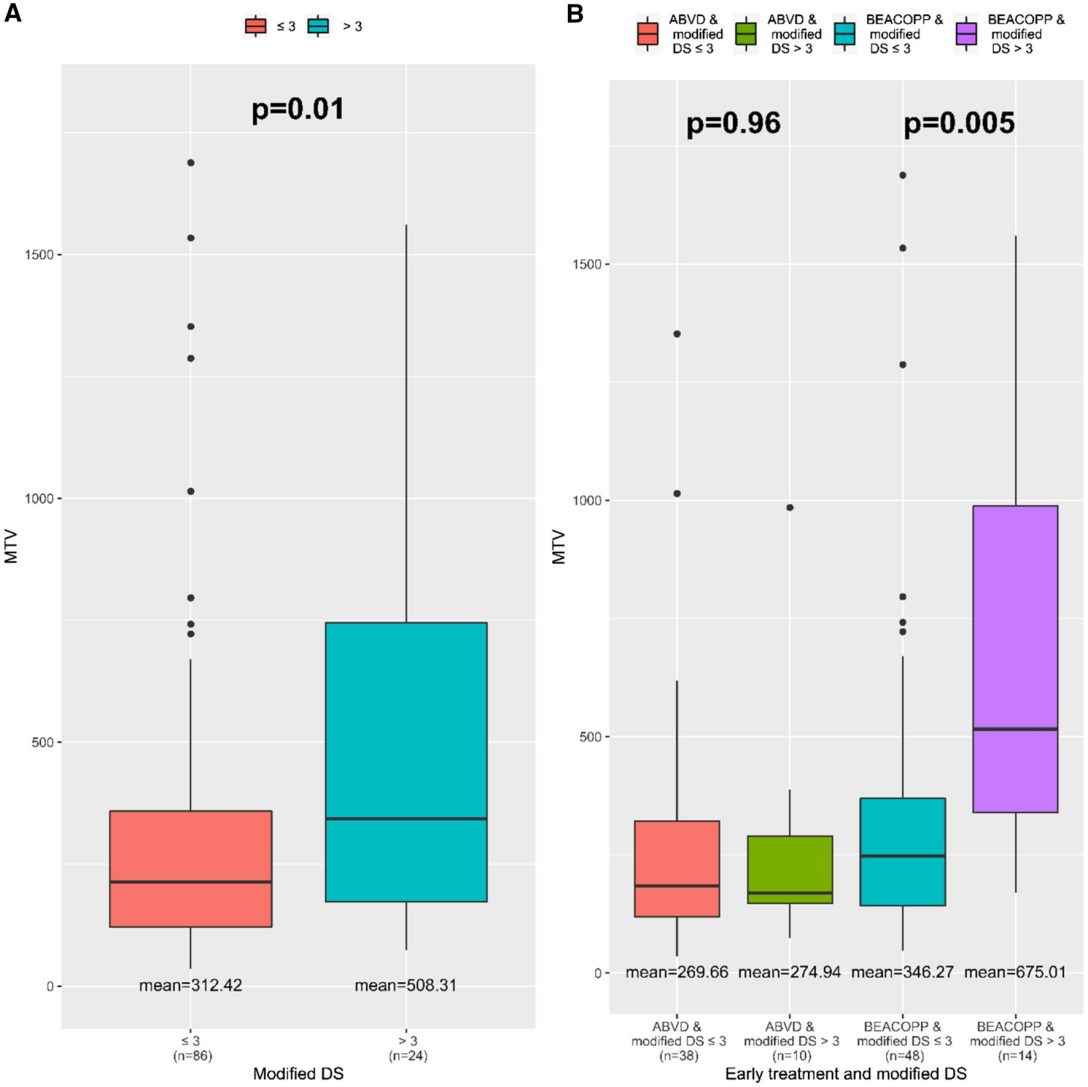
Prognostic value of total metabolic tumour volume (TMTV) for 2-cycle PET scan response according to modified Deauville score.

Of the 110 patients who had PET2, the mean TMTV of 312.42 cm^3^ in the negative PET2 subgroup was significantly lower than the mean TMTV of 508.31 cm^3^ in the positive PET2 subgroup (*p* = 0.01). Among the 57 patients with a high TMTV, 17 (29.8%) had positive PET2 results (Fig. 4).

Similar results were found with the Deauville score.

#### Subgroup analysis

Among the 62 patients treated with BEACOPP, there was a significant difference between the mean TMTV in the negative PET2 subgroup (346.27 cm^3^) and the one in the positive PET2 subgroup (675.01 cm^3^), *p* = 0.005, using the modified Deauville score (Fig. 4) or Deauville score. In contrast, no difference was observed in the subgroup treated with ABVD (n = 48), according to the mean TMTV (*p* = 0.96) (Fig. 4).

### Multivariate analysis

In multivariate analysis using the Cox model and combining TMTV with known initial prognostic factors (IPI score and first treatment received), TMTV < 217 cm^3^ and a first treatment with BEACOPP were associated with a significantly longer PFS (HR 0.43, *p* = 0.02, HR 0.29, and *p* = 0.003, respectively), whereas none of these parameters were associated with a significantly longer OS (Table 2).

**Table 2:** Multivariate analysis.

	Progression-free survival (5 years)				Overall survival (5 years)			
	Coefficient (SE)	HR (95% CI)	*p* value	*p* value (Hochberg correction)	Coefficient (SE)	HR (95% CI)	*p* value	*p* value (Hochberg correction)
**International prognostic score**								
3–7		1				1		
0–2	− 0.11 (0.30)	0.90 (0.49–1.63)	0.72	0.72	− 0.09 (0.49)	0.92 (0.35–2.39)	0.86	0.86
**First line of treatment**								
ABVD		1				1		
BEACOPP	− 1.25 (0.39)	0.29 (0.13–0.61)	0.001	0.003	− 1.44 (0.75)	0.24 (0.05–1.03)	0.05	0.15
**MTV**								
≥ 217 cm^3^		1				1		
< 217 cm^3^	− 0.85 (0.32)	0.43 (0.23–0.80)	0.008	0.02	− 0.91 (0.54)	0.40 (0.14–1.15)	0.09	0.18

### Combined analysis

**Figure 5:**
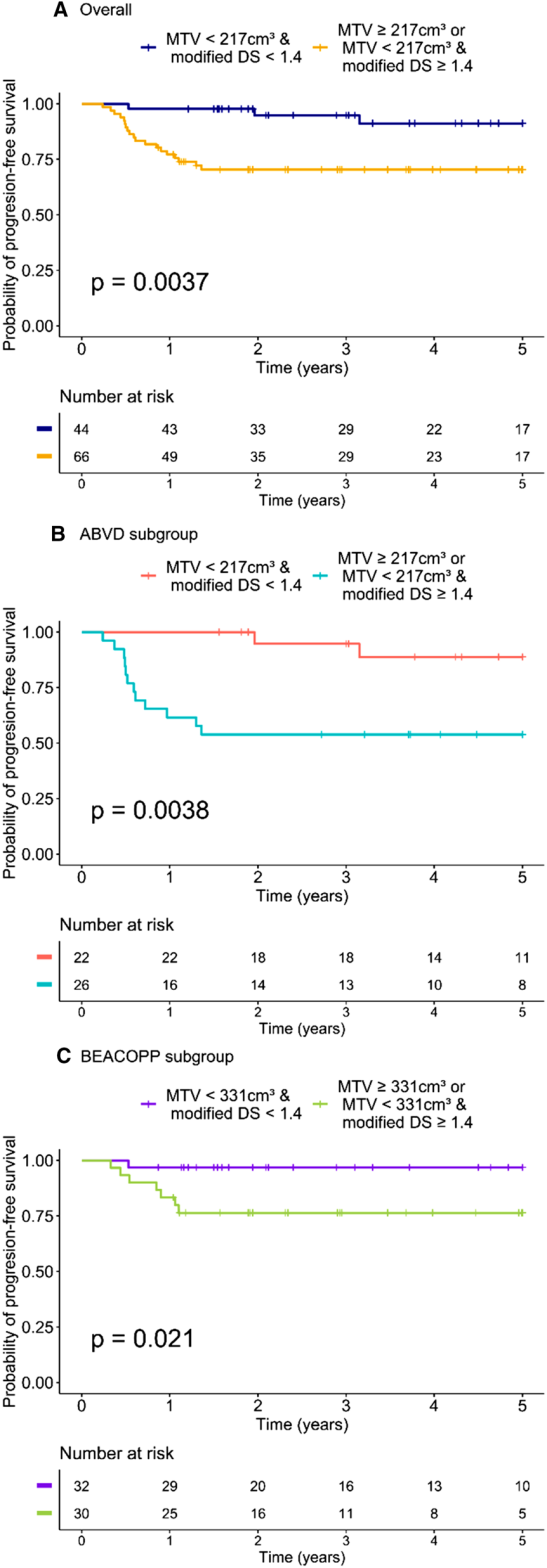
Five-year progression-free survival analysis using two risk categories of patients according to total metabolic tumour volume (TMTV) and modified Deauville score.

Combining the TMTV- and PET2-modified Deauville score allowed us to identify two risk categories. Patients with a TMTV < 217 cm^3^ and a negative PET2 had a significantly higher 5-year PFS than those with a TMTV ≥ 217 cm^3^ or a positive PET2 (*p* = 0.0037). The 44 patients with a low TMTV and a negative PET2 had a significantly better outcome, with a 5-year PFS of 91% vs. 70% for patients with a higher TMTV or positive PET2 (Fig. 5).

#### Subgroup analysis

In the ABVD subgroup, patients with a TMTV < 217 cm^3^ and a negative PET2 had a significantly higher 5-year PFS than those with a TMTV ≥ 217 cm^3^ or a positive PET2 (89% vs. 54%, respectively, *p* = 0.0038). Similar results were found in the BEACOPP subgroup, with patients with a TMTV < 331 cm^3^ and a negative PET2 that had a significantly higher 5-year PFS than those with a TMTV ≥ 331 cm^3^ or a positive PET2 (97% vs. 77%, respectively, *p* = 0.021) (Fig. 5).

## Discussion

To our knowledge, this is the first study to demonstrate the independent prognostic value of baseline TMTV in advanced-stage HL. A high TMTV allows the identification of patients with high risk for HL recurrence in the whole population. The results of multivariable analysis involving TMTV, IPI score, and the first treatment received showed that TMTV remains a baseline prognostic factor for 5-year PFS, in contrast with IPI score. Moreover, the combined analysis of baseline TMTV and PET response after two courses of chemotherapy enabled us to individualise a subgroup of patients with a very low risk of recurrence compared to the others (low TMTV and complete response on PET2).

However, in subgroup analysis, TMTV remains predictive of 5-year PFS only in patients treated with ABVD. In the population of patients treated with BEACOPP, optimal cut-off was higher (331 cm^3^) compared to ABVD subgroup (217 cm3). This is most likely because BEACOPP-treated patients are significantly younger (median age 33 years versus 41 years for the ABVD group) and have a significantly higher TMTV (median 307 cm^3^ versus 217 in the ABVD group). In this subgroup, TMTV is predictive of response after two courses of chemotherapy.

These results are in accordance with those of Mettler et al.^7^, who also showed the predictivity of TMTV on PET2 response but not PFS in a prospective study of 310 patients treated with BEACOPP. As suggested by the authors, this could be explained by a higher complete remission rate with escalated therapy than with anthracycline-based treatment. Furthermore, the high efficacy of BEACOPP after a positive PET2 could mask the prognostic value of TMTV that could be observed in patients receiving ABVD. In our study, only eight patients who were initially treated with BEACOPP relapsed. Of these eight patients, six had a TMTV ≥ 331 cm^3^ (mean, 679 cm^3^) and five had a positive PET2. Therefore, the low number of events probably limited the results obtained.

The prognostic value of TMTV is now well known in diffuse large B-cell lymphoma^16–18^, peripheral T-cell lymphoma^19^, and early stage HL^6^, but remains poorly studied in advanced HL. In 2019, Cottereau et al. showed that in a population of 258 patients with early stage HL from the standard arm of the H10 trial, an initial TMTV of < 147 cm^3^ was predictive of better 5-year PFS and OS^6^. In 2014, Kanoun et al. demonstrated the predictive value of baseline TMTV in terms of 4-year PFS in 59 patients with early- or advanced-stage HL, with a cut-off value of 225 cm^3^^20^. All the patients in these two studies were initially treated with ABVD.

The TMTV values we found were consistent with those reported in other studies on similar subjects. In a preliminary analysis presented at the American Society of Clinical Oncology (ASCO), using the same method of segmentation, Casasnovas et al.^21^ found an optimal cut-off of TMTV of 350 cm^3^ (vs. 331 cm^3^ in our study) in a population of patients with advanced-stage HL treated with BEACOPP. Kanoun et al.^20^ found a median TMTV of 117 cm^3^ (vs. 217 cm^3^ in our study) in a population of patients with early or advanced HL treated with ABVD. This lower value is probably related to the inclusion of early stage patients. However, the predictive cut-off for PFS in this study was very close to ours: 225 vs. 217 cm^3^ in our study.

We chose the segmentation method using the 41% SUVmax threshold to determine the TMTVs, as recommended^22,23^. However, this method does not seem to be the most reproducible^24^, and methods with fixed thresholds may be preferred. In our study, reproducibility of segmentation remained excellent, with an ICC between 0.91 and 0.93, depending on the observers for the measurement of TMTV. This excellent reproducibility can be explained by the fact that a semi-automatic method was used.

Although the prognostic value of TMTV has been demonstrated in several types of lymphoma, TMTV is rarely used in clinical practice. Its measurement is time consuming as each lesion must be segmented individually. To solve this problem, several automatic segmentation methods have been developed in recent years. Among them, those using convolutional neural networks seem the most promising. Currently, they are less reliable than human segmentations, but could potentially allow, in the near future, a reliable estimation of TMTV in a systematic way^25^.

The impact of this new prognostic factor has to be evaluated in patients treated with new drug combinations including agents such as brentuximab vedotin or a checkpoint inhibitor and has to be assessed in prospective clinical trials testing the relevance of adapted therapy depending on TMTV and PET2 response.

Our study has some limitations, including its retrospective nature, the relatively small number of patients included, and the heterogeneity of chemotherapy protocols. In addition, the analysis of response to two courses of treatment was limited by the fact that PET2 was not performed for all patients. However, it highlights the prognostic value of TMTV in advanced HL, which could enable the definition of new groups of patients according to their risk of recurrence. In particular, the use of composite criteria considering PET2 response associated with initial TMTV could be relevant and allow the implementation of a possible protocol of treatment relief for the group of patients with a very good prognosis.

## Conclusion

Baseline TMTV appears to be a useful independent prognostic factor for predicting relapse in advanced stage HL in the ABVD subgroup, with a tendency of survival curves separation in BEACOPP subgroup and could be used to improve risk stratification. However, its use in everyday practice is limited owing to the multiplicity of segmentation methods and its time-consuming nature. Further prospective investigations are needed to evaluate the benefits of including baseline TMTV as a factor in determining treatment regimen.

## References

[1] Ansell SM (2018). Hodgkin lymphoma: 2018 update on diagnosis, risk-stratification, and management. Am. J. Hematol..

[2] Tubiana M, Henry-Amar M, Carde P, Burgers JM, Hayat M, Van der Schueren E, Noordijk EM, Tanguy A, Meerwaldt JH, Thomas J (1989). Toward comprehensive management tailored to prognostic factors of patients with clinical stages I and II in Hodgkin’s disease. The EORTC Lymphoma Group controlled clinical trials: 1964–1987. Blood.

[3] Hasenclever D, Diehl V (1998). A prognostic score for advanced Hodgkin’s disease. International Prognostic Factors Project on Advanced Hodgkin’s Disease. N. Engl. J. Med..

[4] Casasnovas R-O, Bouabdallah R, Brice P, Lazarovici J, Ghesquieres H, Stamatoullas A, Dupuis J, Gac A-C, Gastinne T, Joly B (2019). PET-adapted treatment for newly diagnosed advanced Hodgkin lymphoma (AHL2011): a randomised, multicentre, non-inferiority, phase 3 study. Lancet Oncol..

[5] André MPE, Girinsky T, Federico M, Reman O, Fortpied C, Gotti M, Casasnovas O, Brice P, van der Maazen R, Re A (2017). Early positron emission tomography response-adapted treatment in stage I and II Hodgkin lymphoma: Final results of the randomized EORTC/LYSA/FIL H10 trial. J. Clin. Oncol..

[6] Cottereau A-S, Versari A, Loft A, Casasnovas O, Bellei M, Ricci R, Bardet S, Castagnoli A, Brice P, Raemaekers J (2018). Prognostic value of baseline metabolic tumor volume in early-stage Hodgkin lymphoma in the standard arm of the H10 trial. Blood.

[7] Mettler J, Müller H, Voltin C-A, Baues C, Klaeser B, Moccia A, Borchmann P, Engert A, Kuhnert G, Drzezga AE (2018). Metabolic tumour volume for response prediction in advanced-stage hodgkin lymphoma. J. Nucl. Med..

[8] Kanoun S, Tal I, Berriolo-Riedinger A, Rossi C, Riedinger J-M, Vrigneaud J-M, Legrand L, Humbert O, Casasnovas O, Brunotte F (2015). Influence of software tool and methodological aspects of total metabolic tumor volume calculation on baseline [18F]FDG PET to predict survival in hodgkin lymphoma. PLoS ONE.

[9] Barrington SF, Meignan M (2019). Time to prepare for risk adaptation in lymphoma by standardizing measurement of metabolic tumor burden. J. Nucl. Med..

[10] R: The R Project for Statistical Computing. Available at: https://www.r-project.org/ [Accessed June 30, 2021].

[11] Shrout PE, Fleiss JL (1979). Intraclass correlations: uses in assessing rater reliability. Psychol. Bull..

[12] Davison AC. Bootstrap Methods and their Application.47.

[13] Altman DG, De Stavola BL, Love SB, Stepniewska KA (1995). Review of survival analyses published in cancer journals. Br. J. Cancer.

[14] Hochberg Y (1988). A Sharper Bonferroni procedure for multiple tests of significance. Biometrika.

[15] Koo TK, Li MY (2016). A guideline of selecting and reporting intraclass correlation coefficients for reliability research. J. Chiropr. Med..

[16] Song M-K, Chung J-S, Shin H-J, Lee S-M, Lee S-E, Lee H-S, Lee G-W, Kim S-J, Lee S-M, Chung D-S (2012). Clinical significance of metabolic tumor volume by PET/CT in stages II and III of diffuse large B cell lymphoma without extranodal site involvement. Ann. Hematol..

[17] Chang C-C, Cho S-F, Chuang Y-W, Lin C-Y, Chang S-M, Hsu W-L, Huang Y-F (2017). Prognostic significance of total metabolic tumor volume on 18F-fluorodeoxyglucose positron emission tomography/ computed tomography in patients with diffuse large B-cell lymphoma receiving rituximab-containing chemotherapy. Oncotarget.

[18] Toledano MN, Desbordes P, Banjar A, Gardin I, Vera P, Ruminy P, Jardin F, Tilly H, Becker S (2018). Combination of baseline FDG PET/CT total metabolic tumour volume and gene expression profile have a robust predictive value in patients with diffuse large B-cell lymphoma. Eur. J. Nucl. Med. Mol. Imaging.

[19] Cottereau AS, Becker S, Broussais F, Casasnovas O, Kanoun S, Roques M, Charrier N, Bertrand S, Delarue R, Bonnet C (2016). Prognostic value of baseline total metabolic tumor volume (TMTV0) measured on FDG-PET/CT in patients with peripheral T-cell lymphoma (PTCL). Ann. Oncol..

[20] Kanoun S, Rossi C, Berriolo-Riedinger A, Dygai-Cochet I, Cochet A, Humbert O, Toubeau M, Ferrant E, Brunotte F, Casasnovas R-O (2014). Baseline metabolic tumour volume is an independent prognostic factor in Hodgkin lymphoma. Eur. J. Nucl. Med. Mol. Imaging.

[21] Casasnovas R-O, Kanoun S, Tal I, Cottereau A-S, Edeline V, Brice P, Bouabdallah R, Salles GA, Stamatoullas A, Dupuis J (2016). Baseline total metabolic volume (TMTV) to predict the outcome of patients with advanced Hodgkin lymphoma (HL) enrolled in the AHL2011 LYSA trial. JCO.

[22] Boellaard R, O’Doherty MJ, Weber WA, Mottaghy FM, Lonsdale MN, Stroobants SG, Oyen WJG, Kotzerke J, Hoekstra OS, Pruim J (2010). FDG PET and PET/CT: EANM procedure guidelines for tumour PET imaging: version 1.0. Eur. J. Nucl. Med. Mol. Imaging.

[23] Meignan M, Sasanelli M, Casasnovas RO, Luminari S, Fioroni F, Coriani C, Masset H, Itti E, Gobbi PG, Merli F (2014). Metabolic tumour volumes measured at staging in lymphoma: methodological evaluation on phantom experiments and patients. Eur. J. Nucl. Med. Mol. Imaging.

[24] Eude F, Toledano MN, Vera P, Tilly H, Mihailescu S-D, Becker S (2021). Reproducibility of baseline tumour metabolic volume measurements in diffuse large B-Cell LymphomA: Is there a superior method?. Metabolites.

[25] Pinochet P, Eude F, Becker S, Shah V, Sibille L, Toledano MN, Modzelewski R, Vera P, Decazes P (2021). Evaluation of an automatic classification algorithm using convolutional neural networks in oncological positron emission tomography. Front. Med. (Lausanne).

